# Metastasis in penile corpus cavernosum from esophageal squamous carcinoma after curative resection: a case report

**DOI:** 10.1186/s12885-019-5356-3

**Published:** 2019-02-20

**Authors:** Lingmin Song, Yangmin Wang, Guobin Weng

**Affiliations:** 1 Department of Urology, Ningbo Urology & Nephrology Hospital, Ningbo, 315192 Zhejiang China; 2 Department of Urology, Lanzhou General Hospital, PLA, Lanzhou, Gansu China

**Keywords:** Penile metastasis, Esophageal carcinoma, Palliative therapy, Case report

## Abstract

**Background:**

Metastasis in penile corpus cavernosum from esophageal squamous carcinoma is a rare but fatal disease, which was reported in cases without series studies.

**Case presentation:**

An 84-year-old male smoker, who had a history of curative resection of esophageal squamous carcinoma 12 months before, presented with aggressive dysuria and penis pain for 1 month. Ultrasonic guided biopsy diagnosed metastatic squamous carcinoma from the primary in the esophagus. The accurately modulated conformal radiotherapy and non-steroidal antiinflammatory drugs achieved to alleviate the penis pain temporarily. But the disease progressed and disseminated in a short period. He died of multiple metastases and cancer cachexia in 4 months.

**Conclusions:**

Primary esophageal cancer metastasis to penile corpus cavernosum refers to short onset time of metastasis, extensive dissemination, bad response to treatment and poor prognosis. Palliative therapy to patients with the disease could achieve temporary local symptom relief, but not prolong survival time. More research is necessary to understand the underlying mechanism of esophagheal metastasis.

## Background

Esophageal cancer is one of the most deadly cancers worldwide, with extremely aggressive nature and 5-year survival of 15–25%, and the main metastatic organs are reported to be liver, lung, bone and brain [1]. Histologic types include squamous carcinoma, adenocarcinoma and undifferentiated carcinoma [2]. Despite the abundant blood supply, penile metastatic cancer, with a poor prognosis of 10-month median survival time, is uncommonly reported no more than 500 cases hitherto [3]. Among these cases, 69% of penile metastases were primarily from urogenital cancers and 19% from gastrointestinal caner [4]. Since Gupta NM reported the first case of penile metastasis from esophageal cancer in 1989, only 9 cases were reported worldwide until now [5–13], which were all case reports without systematical study because of the rarity. The present study reports a new case about the clinical characteristic and management of penile metastases from esophageal cancer. Furthermore, we reviewed all the case reports to provide a summary of the clinical symptoms, treatments, survival and intended to speculate about the possible risk factors, research targets for this disease.

## Case presentation

**Fig. 1:**
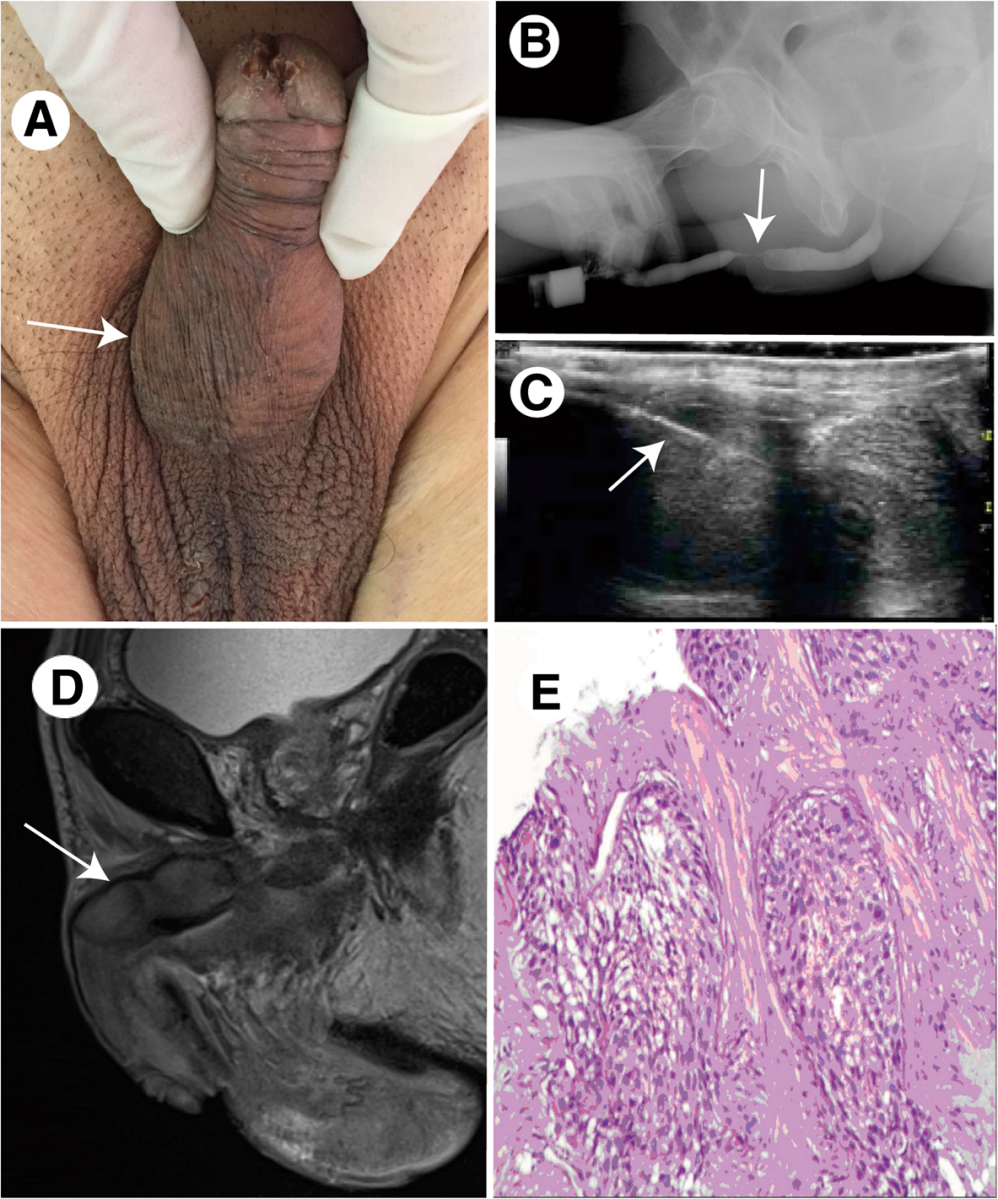
Urethrostenosis Caused by Metastasis in Penile Corpus Cavernosum from Esophageal Squamous Carcinoma. **a**: gross appearance of the penis and the palpable hard nodules (arrow); **b**: retrograde urethrography showed the secondary urethrostenosis (arrow); **c**: ultrasonic guided biopsy from one of the nodules, the arrow showed the needle passage of operating biopsy; **d**: the mass in the right penile corpus cavernosum confirmed by MRI-T2 weighted phase (arrow); **e**: pathological diagnosis of the biopsy detected the metastatic squamous carcinoma (HE stain: × 40)

In September 2017, an 84-year-old male smoker, who had a history of curative resection of esophageal squamous carcinoma (pT3N0M0, phase IIA, moderately differentiated) 12 months before, presented with aggressive dysuria and penis pain for 1 month. He was in good performance status (ECOG = 1) with stage 2 hypertension for 30 years, which was under control by regular Nifedipine GITS. His physical examination revealed roughly normal appearance of the penis but several smooth, hard, fixed nodules (diameter from 0.5 cm to 2.5 cm) in the right penile corpus cavernosum, which compressed the penis urethra(Fig. 1a). MRI pelvis protocol scanning confirmed these masses, but did not detect any obvious metastasis in pelvic lymph nodes, bones or lumber, sacral vetebras,(Fig. 1d). Gastroscopy with biopsy at the anastomosis detected no sign of local recurrence, and there was no radiographic evidence of pulmonary or mediastinal metastases by CT scan. After the failure of urethroscopy, retrograde urethrography showed a 2 cm-length urethrostenosis about 5 cm proximal to external orifice(Fig. 1b), and he was catheterized (F12, Foley) in case of acute urinary retention. Ultrasonic guided biopsy (Fig. 1c) from one of the nodules diagnosed metastatic squamous carcinoma from the primary in the esophagus(Fig. 1e). IHC revealed positive expression of CK8/18, CK5/6, P40, while negative expression of CK7, CK20. He refused positron emission tomography scan with CT, penectomy or chemotherapy. Then after paracentetic suprapubic cystostomy, we offered him accurately modulated conformal radiotherapy (total radiation absorbed dose: 6000 cGy/30 times) and non-steroidal antiinflammatory drugs (NSAIDs) to alleviate the penis pain. But 4 weeks later, the hard nodules in penile corpus cavernosum progressed; furthermore he developed severe back pain. MRI detected metastasis in the 4th and 5th lumber vertebrae. Since he still rejected further chemotherapy or radiotherapy, we treated him palliatively with paraspinal nerve block and three ladder analgesic programs of cancer to temporarily relieve the pain. After 10 weeks, he presented to us with cough, chest pain and recurrent dysphagia. CT scan revealed pulmonary infection, metastasis in both lungs and suspicious local recurrence in esophagus. He was discharged when pulmonary infection was cured, and the therapeutic regime turned to hospice care. In January 2018, he died of multiple metastases and cancer cachexia.

### Literature review

We performed a search using PubMed and Chinese National Knowledge Infrastructure (CNKI), which offers medical literature research in China. As mentioned above, to the best of our knowledge only 9 cases of penile metastasis from esophageal cancer have been reported from 1989 to 2018. From these 10 cases, including the present and previous reports, we analyzed the clinical feature of the primary (Table 1) and the metastatic cancer (Table 2) in order to get some information for further study about this disease.

**Table 1:** Clinical characteristics and treatment of primary cancer

Author	Age (year)	Primary treatment	Pathology	Stage	Postoperative Therapy
Gupta N M	40	Radiotherapy	Poorly differentiated squamous cell carcinoma	Can’t evaluate	–
Pai A	51	Curative resection	Moderately differentiated squamous carcinoma	pT3pN1M0	Adjuvant radiotherapy
Zou C	61	Curative resection	Moderately differentiated squamous cell carcinoma	pT3pN1M0	Radiotherapy plus chemotherapy
Numakura K	68	Curative resection	Poorly differentiated squamous cell carcinoma	pT3pN0M0	–
Kobayashi Y	61	Curative resection	Well differentiated squamous cell carcinoma	pT3pN0M0	Adjuvant chemotherapy
López-Aramburu MA	57	Chemotherapy	Squamous cell carcinoma	cT3NxM1	–
Morán PE	53	Chemotherapy plus Radiotherapy	Moderately differentiated squamous cell carcinoma	cT4N1M1	–
Li XM	57	Lower esophagectomy	Squamous cell carcinoma	Can’t evaluate	–
Tang ZJ	49	Middle esophagectomy	Moderately differentiated squamous cell carcinoma	Can’t evaluate	–
Song LM	84	Curative resection	Moderately differentiated squamous cell carcinoma	pT3N0M0	–

**Table 2:** Treatment, pathological findings, and follow-up of 9 patients with metastatic penile cancer

Author	Time of penile metastasis	Clinical manifestation	Metastatic site	Other metastasis simultaneously	Diagnosis method	Pathology	Treatment of metastasis	Survival time
Gupta NM	1 week	Painful nodules	Dorsal shaft	Not mentioned	Open biopsy	Squamous cell carcinoma	Polydrug chemotherapy	6 weeks
Pai A	9 months	Painful nodules	Full length	Lymph node in left hilar, subcarinal and left supraclavicular	Fine needle aspiration	Squamous cell carcinoma	Palliative chemotherapy	alive in 6 months
Zou C	6 months	Painless mass	Shaft	Right thigh; lymph nodes in left neck	Excisional biopsy	Moderated differentiated squamous cell carcinoma	Resection of penile mass; Palliative external beam radiation	5 months
Numakura K	24+ months	Dysuria, Penile pain Necrosis in glans	Full length	Brain, liver and eyes	Excisional biopsy	Squamous cell carcinoma	Total penectomy, chemotherapy	3 months
Kobayashi Y	2 months	Painless mass	Glans and shaft	Abdominal lymph nodes and spleen	Open biopsy	Squamous cell carcinoma	Chemotherapy	5 months
López-Aramburu MA	Simultaneously penile metastasis was the first symptom	Pain, Necrosis in glans	Glans and distal shaft	Right humerus	Excisional biopsy	Squamous cell carcinoma	Partial penectomy	1 month
Morán PE	24+ months	Painless mass, Dysuria	Glans and shaft	Pelvic lymph node	Open biopsy	Squamous cell carcinoma	Chemotherapy plus radiotherapy	Withdrawing, loss to follow up
Li XM	9 months	Priapism, Painless nodle, Dysuria	Shaft	Not mentioned	Excisional biopsy	Squamous cell carcinoma	Total penectomy	10 months
Tang ZJ	4 months	Priapism	Shaft	Not mentioned	Excisional biopsy	Moderately differentiated squamous cell carcinoma	Total penectomy	Not mentioned
Song LM	12 months	Dysuria Painful nodules	Right shaft	Not detected	Ultrasonic guided biopsy	Moderately differentiated squamous cell carcinoma	Radiotherapy	4 months

The average age was 58.1 ± 3.8 years, ranging from 40 to 84 years. Concerning the treatment to primary cancer, curative resection was conducted in 5 cases, partial esophagectomy in 2, chemotherapy and/or radiotherapy in 3. The pathological diagnoses of these primary cancers were exclusively squamous cell carcinoma, of which moderated differentiated account for 50% (5/10). The TNM stages of the primary cancer were pathological or clinical T3N0M0 to T4N1M1, which indicated the primary cancers were advanced, and metastatic when admitted in 4 cases. The time of penile metastasis since primary cancer diagnosed varied a lot, and the mean time was 9.3 ± 2.7 months, ranging from concurrent to more than 24 months. The clinical manifestation included painless (4/10) or painful mass (3/10), necrosis in glans with pain (2/10), dysuria (4/10), and priapism(2/10). The metastatic site could locate in any portion or the full length of penile corpus cavernosum, but none reported in cavernous spongiosum, skin or foreskin. Furthermore, metastases in other organ or lymph node were reported simultaneously in 6 cases. The present case reported the penile metastasis ad the first site and successively metastasis to other organs in 3 months The prognosis was dismal regardless of the methods of treatment to metastases, since the survival time was 5.4 ± 1.4 months (ranging from 1 month to 12 months).

## Discussion and conclusions

The present report and analysis of literature review reveal that metastasis in penile corpus cavernosum from esophageal squamous carcinoma is a rare disease with a survival time of less than 12 months after diagnosed and poor treatment response. Since this disease is only reported in case reports, there might rarely be reliable evidence from lager data of prospective or retrospective series studied. However, a little more information can be gathered from these cases available. Thereupon, we get conscious of some characteristics of this disease: the primary cancers were exclusively squamous cell carcinoma and local advanced, even metastatic when admitted in a few cases; the metastatic site exclusively located in penile corpus cavernosum, but not in cavernous spongiosum, skin or foreskin; metastasis to other sites simultaneously were detected in most cases, and progressed rapidly with a poor prognosis.

It is widely accepted that the possible mechanisms of metastasis include direct invasion, hematogenous or lymphatic dissemination, iatrogenic spread [3]. Obviously, anatomic proximity is necessary for direct invasion, such as large metastatic mass in the root of penis from the prostate and bladder [14] Penile metastasis from genitourinary system and colon/rectum via pelvic venous plexus and dorsal penile vein was presumed by Abeshouse and Kumer with the evidence of presentation of cancer thrombi in vasculature [15, 16]. While Paquin proposed retrograde lymphatic metastasis to penis might basically locate in the skin, foreskin, because of the abundant lymph supply and widely connection of the penis with pelvic organs via the iliac lymph nodes [14]. Urethra or cavernous spongiosum might be involved by transurethral resection of the prostate or bladder cancer via iatrogenic spread [17]. Because of the anatomical distance, it seems obvious that esophageal cancer does not metastasis via direct invasion, venous or lymphatic dissemination, and iatrogenic spread to the penis. Therefore, we speculate arterial dissemination be the metastatic pathway, which simultaneously indicates the progression and widespread dissemination of the primary cancer.

The treatment, including surgical excision, urinary diversion, radiotherapy, chemotherapy and the combination of these, is usually palliative and relieve local symptom temporarily, but may not delay the progression or prolong survival time. The poor response to treatment lead to the dismal prognosis no more than 6-month’s median survival time not only in penile metastasis from esophageal cancer but also from other primary cancers [3, 9]. However, little is known about the exact mechanism or molecular biology feature of penile metastasis especially from esophageal cancer, which needs to be verified by further research.

Primary esophageal cancer metastasis to penile corpus cavernosum is a rare but fatal condition, which refers to short onset time of metastasis, extensive dissemination, bad response to treatment and poor prognosis. Palliative therapy to patients with the disease could achieve temporary local symptom relief, but not prolong survival time. The rarity of the disease limits clinical study and biochemistry research on it, as well as our understanding of the underlying mechanisms of metastasis for effective treatment and better prognosis.

## Abbreviations

cGy: Centigray
CK: Cytokeratin
CNKI: Chinese National Knowledge Infrastructure
CT: Computed Tomography
ECOG: Eastern Cooperative Oncology Group
IHC: Immunohistochemistry
MRI: Magnetic Resonance Imaging
NSAIDs: Non-steroidal antiinflammatory drugs
